# Boiling sheep liver or lung for 30 minutes is necessary and sufficient to kill *Echinococcus granulosus* protoscoleces in hydatid cysts[Fn FN1]

**DOI:** 10.1051/parasite/2014064

**Published:** 2014-12-02

**Authors:** Jun Li, Chuanchuan Wu, Hui Wang, Huanyuan Liu, Dominique A. Vuitton, Hao Wen, Wenbao Zhang

**Affiliations:** 1 State Key Laboratory Incubation Base for Xinjiang Major Diseases Research and Xinjiang Key Laboratory of Echinococcosis, The First Affiliated Hospital of Xinjiang Medical University Urumqi, Xinjiang China; 2 Department Immunology, School of Preclinical Medicine, Xinjiang Medical University Urumqi, Xinjiang China; 3 College of Veterinary Medicine, Xinjiang Agricultural University Urumqi, Xinjiang China; 4 WHO-Collaborating Centre for the Prevention and Treatment of Human Echinococcosis, University of Franche-Comté and University Hospital Besançon Franche-Comté France

**Keywords:** *Echinococcus granulosus*, Cystic echinococcosis, Hydatid cyst, Control measures, Community health

## Abstract

Proper disposal of carcasses and offal after home slaughter is difficult in poor and remote communities and therefore dogs readily have access to hydatid cysts containing offal from livestock, thus completing the parasite cycle of *Echinococcus granulosus* and putting communities at risk of cystic echinococcosis. Boiling livers and lungs which contain hydatid cysts could be a simple, efficient and energy- and time-saving way to kill the infectious protoscoleces. The aim of this study was to provide precise practical recommendations to livestock owners. Our results show that boiling the whole sheep liver and/or lung, with single or multiple hydatid cysts, for 30 min is necessary and sufficient to kill *E. granulosus* protoscoleces in hydatid cysts. Advertising on this simple rule in at-risk communities would be an efficient and cheap complement to other veterinary public health operations to control cystic echinococcosis.

## Abstract (zh)

采用简便易行的方法在贫困和偏远的农牧区处理患有包虫病家畜的内脏是防止犬感染细粒棘球绦虫和控制包虫病传播的关键措施。煮沸法应该是杀灭病畜肝肺内原头蚴的一种简便易行和节能省时的方法。本研究的目的是给家畜饲养者提供准确的杀死肝肺内细粒棘球绦虫原头蚴的时间和方法。结果表明，用煮沸的方法杀死含有单个或多个包虫囊羊肝或羊肺内的原头蚴需要煮沸30分钟的时间。因此建议在包虫病流行的农牧区宣传和推广这一简单而经济有效的方法切断病原循环链, 控制囊型包虫病。

## Abstract (ar)

التخلص السليم من جثث و أحشاء الذبائح بعد ذبح الماشية في المنازل يعد من الصعوبات في المجتمعات المحلية الفقيرة و / أو النائية عن المسالخ المنظمة ؛ لذا، يسهل للكلاب الوصول إلى الفضلات الحاوية على الكيسة العدارية وبالتالي الى استكمال دورة طفيليات المشوكة الحبيبية مما يعرض المجتمعات المحلية لداء الكيسات العدارية. ان غليان كبد ورئتي الذبائح التي تحتوي على الكيسات العدارية قد تكون الوسيلة البسيطة والفعالة التي توفر الوقت و الطاقة لقتل الرؤيسات البدئية المعدية . ان الهدف من هذه الدراسة هو تقديم توصيات عملية و دقيقة لمربي المواشي. نتائجنا تظهر أن غليان الكبد و / أو الرئتين الحاوية على الكيسات العدارية ،المنفردة او المتعددة، لمدة 30 دقيقة هو ضروري وكاف لقتل جميع الرؤيسات البدئية للـمُشْوِكات الحبيبية الموجودة في الكيسات العدارية. ان نشر هذه القاعدة البسيطة في المجتمعات المحلية سيكون بمثابة اجراء مكمل ، فعال وغير مكلف لإجراءات أخرى في الصحة العامة البيطرية للسيطرة على داء الكيسات العدارية.

## Abstract (fr)

Une gestion appropriée des carcasses et des viscères après abattage familial des animaux d’élevage est difficile dans les communautés pauvres et/ou éloignées d’abattoirs organisés. De ce fait, les chiens ont facilement accès aux abats qui contiennent des kystes hydatiques; ils complètent le cycle parasitaire d’*Echinococcus granulosus* et sont à l’origine des cas d’échinococcose kystique dans les communautés. L’ébullition des foies et/ou poumons qui contiennent des kystes hydatiques pourrait être une méthode simple, efficace et peu coûteuse en temps et en énergie, pour détruire les protoscolex responsables de la contamination. L’objectif de cette étude était de donner des règles précises et pratiques aux éleveurs. Nos résultats montrent que bouillir les foies ou poumons contenant des kystes hydatiques pendant 30 minutes est nécessaire et suffisant pour tuer tous les protoscolex d’*Echinococcus granulosus* présents dans ces kystes. La diffusion de cette règle simple dans les communautés serait un complément efficace et peu coûteux aux autres mesures de santé publique vétérinaire pour contrôler l’échinococcose kystique.

## Introduction

Cystic echinococcosis (CE) is a cosmopolitan zoonosis, and its control, albeit theoretically simple, remains practically difficult in most of the endemic areas. The disease is caused by the larval stage of the dog tapeworm *Echinococcus granulosus* [10]. The parasite needs two hosts, a definitive host such as a dog (or a wolf or other carnivores in wildlife), and an intermediate host such as domestic livestock, sheep, goat, cattle, camel or wild herbivores, to complete its life-cycle. Human beings can be accidently infected, but are not involved in the life-cycle. Dogs and other final hosts become infected by feeding on fertile hydatid cysts; these cysts contain viable protoscoleces (PSCs), i.e. young tapeworms. These PSCs develop into mature adult tapeworms in the small intestines of final hosts and each of them produces a gravid segment (proglotids) filled with about 700 eggs. Eggs are released into the open via faeces. Intermediate hosts acquire infection through ingestion of eggs from contaminated vegetation, water or soil. Infections in human beings are favoured through an unhygienic lifestyle, lack of non-contaminated water sources and close man-dog contact. Through digestion in the small intestine, larvae (oncospheres) are freed from the eggs, penetrate actively through the intestinal wall and are carried by the bloodstream into body organs with preference for the liver and lungs, where they develop into cysts and produce hundreds or thousands of PSCs which again need to be eaten by dogs or other definitive hosts to complete the cycle. Interruption of the parasite cycle can thus be done through regular (every 4–6 weeks) mass de-worming of dogs with praziquantel and/or thorough meat inspection by authorised personnel and appropriate offal disposal. Such measures have contributed to a decrease of CE in developed countries such as Western Europe, Australia and New Zealand [2, 4, 5]. These control measures are nearly impossible to implement in the endemic areas where transhumant or nomadic life is common, in sedentary communities widely dispersed in large areas and/or in regions with poor public infrastructure. The usual recommendations given to the farmers/livestock keepers in such communities include offal incineration (burning) or burial. Most often, these measures are not followed, inefficient or even simply impossible because of local constraints. Boiling livers and lungs which contain hydatid cysts could be a simpler, more practical and energy- and time-saving way to kill the pathogenic PSCs. There are however no data available on the optimal time of boiling of viscera containing hydatid cysts to ensure the destruction of the PSCs. The aim of this study was to provide precise practical recommendations to animal owners.

## Materials, methods and results

The sheep livers and lungs were collected from abattoirs in Urumqi, Xinjiang Uyghur Autonomous Region, north-western China. To check the viability of PSCs before boiling the infected viscera, PSCs were removed from three cysts, pooled and stained with 0.1% methylene blue as described previously [9]. After 30 s of staining, PSCs were observed and counted under light microscope. The dead PSCs were stained in blue and the live PSCs remained unstained. To confirm that methylene blue staining could be used for determining the viability of PSCs after liver or lung boiling, we isolated PSCs from hydatid cysts and boiled them directly in water: after 5 min of boiling all the PSCs were stained (data not shown), indicating that methylene blue staining was a reliable method for identifying dead/alive PSCs after this thermal treatment.

To determine the optimal time necessary for killing PSCs in hydatid cysts by boiling the whole liver, we boiled cyst-containing sheep livers in water at 98 °C for 5, 10, 20 and 30 min, respectively. The study was done in triplicate for each time point. From each boiled liver, PSCs were collected, pooled and stained with 0.1% methylene blue; at least 500 PSCs from each infected and boiled liver were counted.

**Figure 1.**
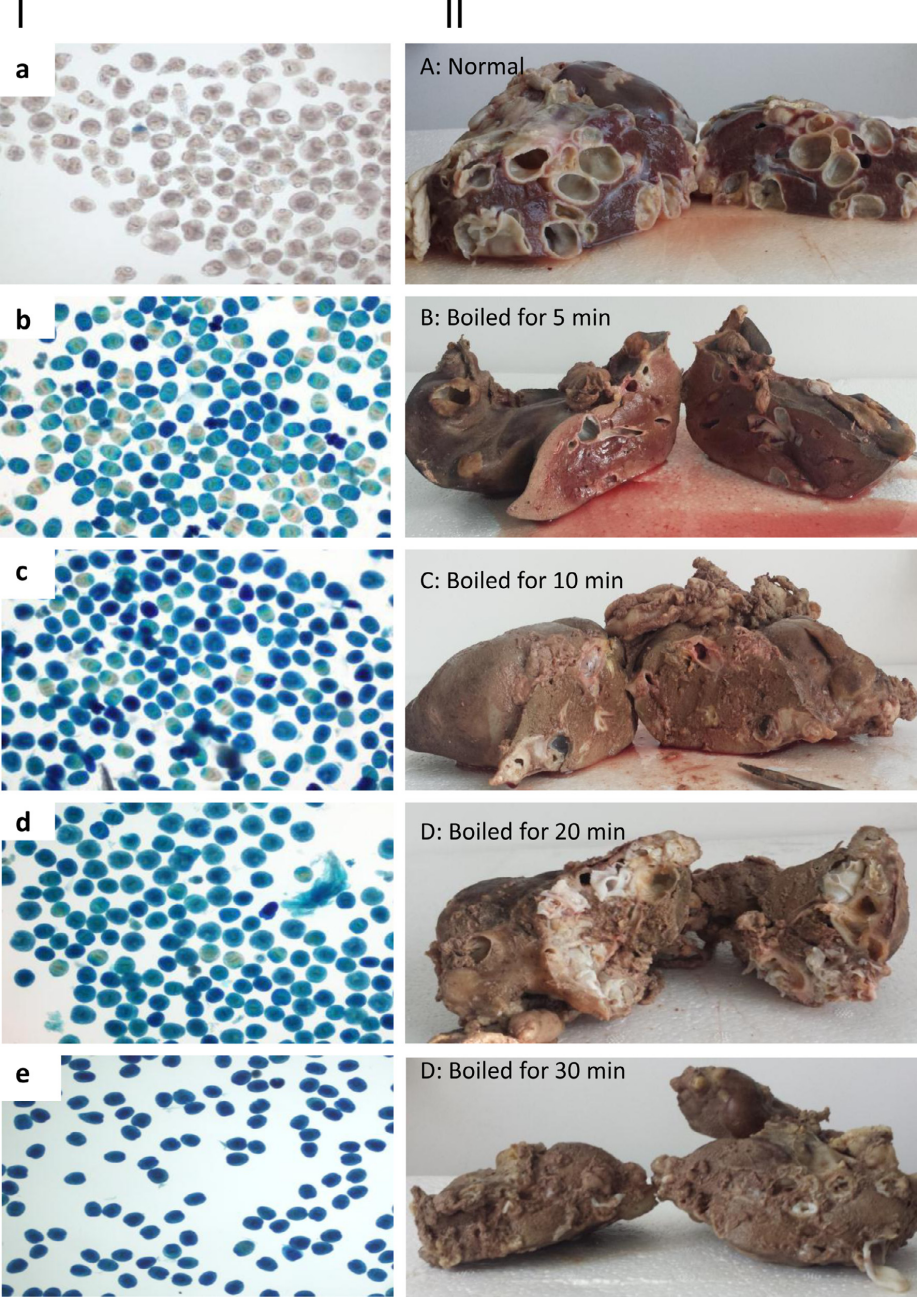
Efficacy of killing *Echinococcus granulosus* protoscoleces (PSCs) by boiling sheep livers containing hydatid cysts. Panel I: (a) normal PSCs were collected from cysts before boiling; (b–e) PSCs were collected from sheep livers boiled for 5, 10, 20 and 30 min, respectively. Panel II: (A) a non-boiled sheep liver containing *E. granulosus* cysts; (B–E) livers boiled for 5, 10, 20 and 30 min, respectively. PSCs stained in blue are dead PSCs.

An average of 7.8%, 3.3% and 0.8% of PSCs were still alive after boiling infected livers for 5 min, 10 min and 20 min, respectively (Table 1 and Figs. 1-Ib–d). All PSCs were blue, thus dead, after boiling for 30 min, indicating that hydatid cyst-containing livers should be boiled at least for 30 min to make sure that all the parasitic material is destroyed and safe (Fig. 1-Ie).

**Table 1.** Survival of protoscoleces of *Echinococcus granulosus* after boiling sheep livers and lungs containing hydatid cysts.

Boiling time (min)	Survival of PSC (%)	
	In livers	In lungs
0	97% (95%–100%)	100%
5	8.7% (0%–16.8%)	13.44% (0–21%)
10	3.3 (0%–9.7%)	3% (0–5.6%)
20	0.8 (0%–2%)	0%
30	0%	0%

In addition, we examined the change of consistency and colour of the boiled livers after the different boiling times. Figure 1-II shows the gradual change of colour from dark brown when fresh to ochre after boiling for 5–30 min, respectively. After 5 min of boiling, the central area of the livers remained with its fresh original colour, with large quantities of blood-like fluid leaking from this area (Fig. 1-Ib). After 20 min, we still observed some blood-like fluid leaking from the central areas of the liver, indicating that these areas were not completely fixed by the heating process. Liver boiled for 30 min was completely fixed with all colour changed to ochre.

**Figure 2.**
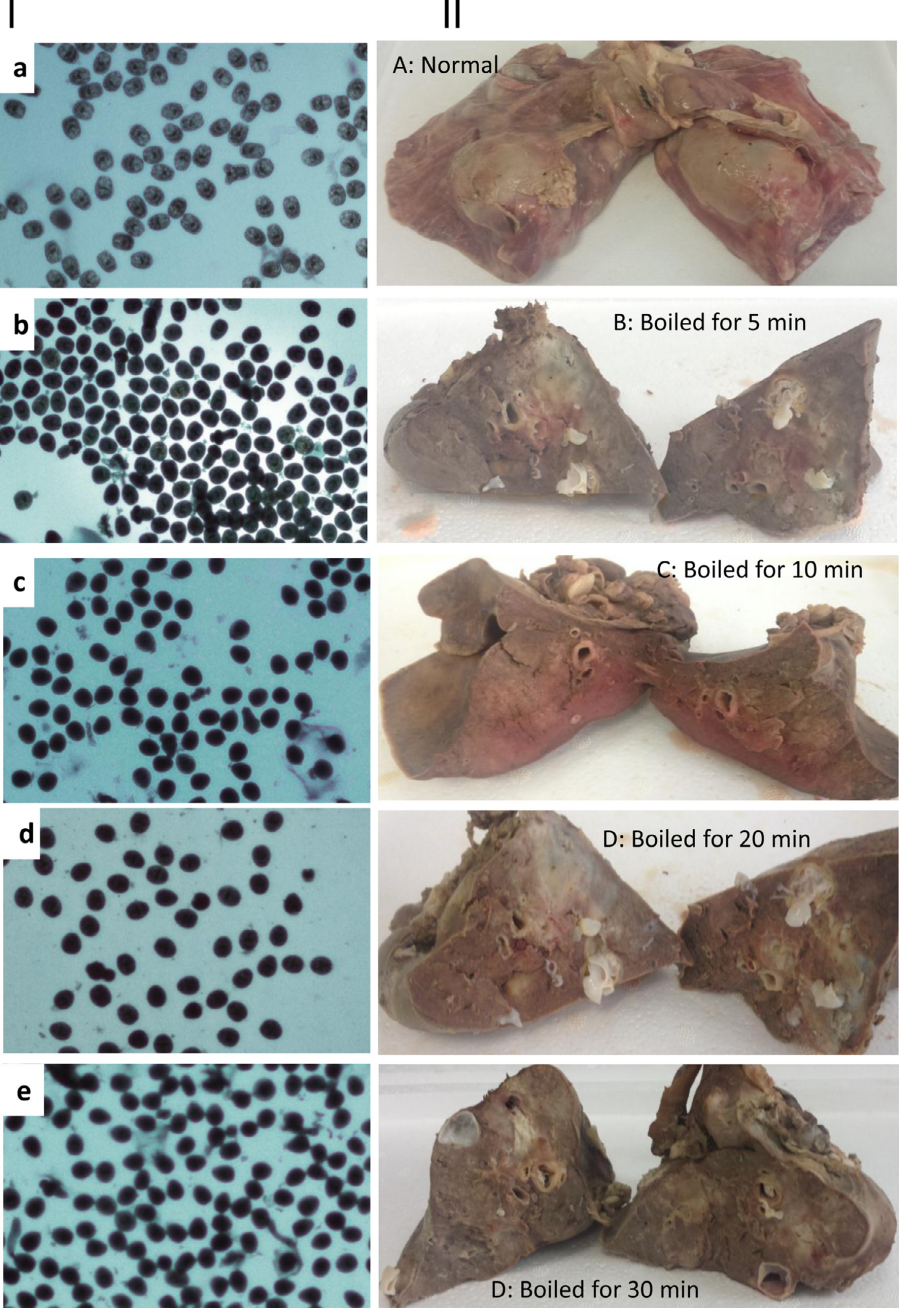
Efficacy of killing *Echinococcus granulosus* protoscoleces (PSCs) by boiling sheep lungs containing hydatid cysts. Panel I: (a) normal PSCs were collected from cysts before boiling; (b–e) PSCs were collected from sheep livers boiled for 5, 10, 20 and 30 min, respectively. Panel II: (A) a non-boiled sheep lung containing *E. granulosus* cysts; (B–E) lungs boiled for 5, 10, 20 and 30 min, respectively. PSCs stained in blue are dead PSCs.

After similarly boiling sheep lungs containing hydatid cysts, the tissue changes were similar to those observed when boiling livers (Fig. 2), but the process needed less time for killing PSCs present in the lungs than in the liver (Table 1).

**Figure 3.**
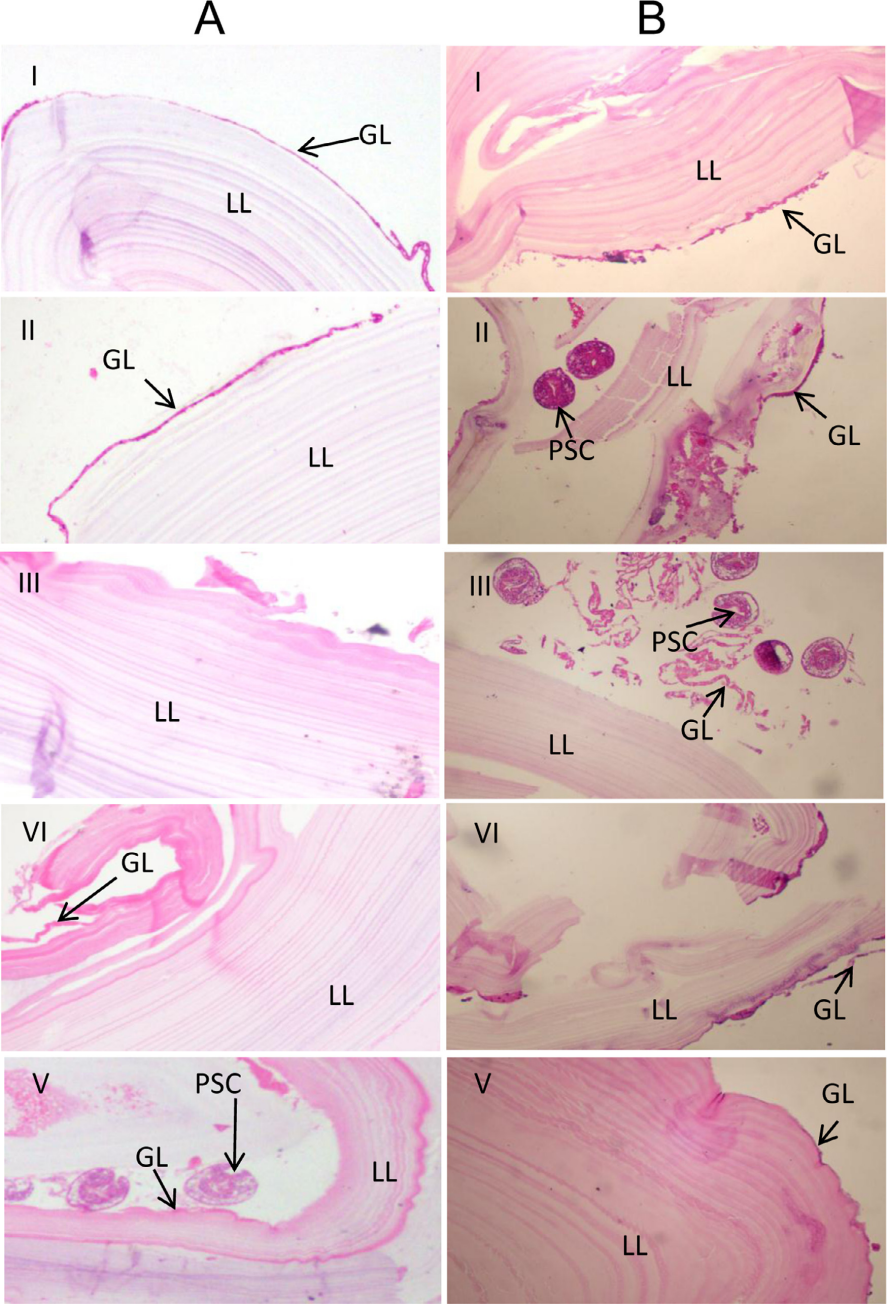
Haematoxylin-eosin (HE) staining of *E. granulosus* cysts from livers (A) and lungs (B) of sheep. I, normal cyst; II–V, cysts boiled for 5, 10, 20 and 30 min, respectively. GL, germinal layer; LL, laminated layer; PSC, protoscolex.

Damage to the structure of the hydatid cyst was histologically confirmed on 4 μm-thick sections of boiled liver and lung hydatid cysts stained with haematoxylin-eosin (Fig. 3).

## Discussion

Our results show that boiling the whole sheep liver and/or lung, with single or multiple hydatid cysts, for 30 min is necessary and sufficient to kill larval *E. granulosus* PSCs in hydatid cysts. CE is a preventable disease [2]. Control measures including preventing dogs from gaining access to dead animals (carcasses) or raw slaughter offal through strict laws on slaughterhouses and meat inspection are well known [6]. However, proper disposal of carcasses and offal after home slaughter is difficult in poor and remote communities and therefore dogs readily have access to hydatid cysts containing organs from livestock. An epidemiological survey in a county of Xinjiang, one of the highly endemic CE areas in China, showed that 84% of households slaughtered sheep and cattle at home and 77% of them fed their dogs with raw animal offal containing hydatid cysts [1]. How to treat the animal offal is problematic in the education of communities. Efficiently managing sick animal offal includes boiling, burning/incineration and burying. It is however surprising to see how little is described in a book as exhaustive as the “WHO/OIE Manual on Echinococcosis in human beings and animals” on the safe disposal of offal [3, 6]. It is difficult to persuade a villager to dig a 1.5 m deep hole to bury animal liver and lungs containing hydatid cysts according to the China national standard “Procedures for biologically harmless handling and treating dead and sick animals and animal products (GB16548-2006)”, as also advised in the above-mentioned “WHO/OIE Manual” [6]. If animal offal is buried less than 60 cm in depth, dogs, foxes and wolves can dig the offal up (personal observation and communication from Prof Wei Li, Qinghai Academy of Animal Science). In addition, the procedure takes time and energy; it faces specific difficulties depending on the nature of the soil and/or particularities of land use; and it usually needs the personal involvement of men (which may be felt detrimental to other activities). Direct burning cannot be performed easily by using the family fire or stove, and is generally avoided because of the smell. In addition, dogs cannot be fed with the ashes. Boiling animal offal for 30 min would be a practical way of sterilising the hydatid cysts present in animal liver or lung in villages or tent/yurt encampments. Even though we observed complete sterilisation of the lung cysts after 20 min of boiling, a single simple message seems preferable. For the livers and lungs from big animals, such as yaks and camels, we suggest cutting the offal into small pieces before the boiling process.

The boiled offal could even be safely given to dogs after the process, which is economically and culturally an advantage. Women are usually in charge of preparing food and boiling meat/soup/vegetables for a certain time is familiar to them; in addition, women in the communities are generally more sensitive to health issues and more easily accessible to health education [7]. Advertising on the simple rule of “boiling livers or lungs of animals for half an hour when they contain cysts” would be an efficient and cheap complement to other control/public health operations in the communities (such as dog dosing with praziquantel or ultrasound mass-screening [8]) in order to decrease the burden of CE in the poorest and least accessible endemic areas.
